# Continuous voluntary community care services for older people in China: Evidence from Wuhu

**DOI:** 10.3389/fpubh.2022.1063156

**Published:** 2023-01-10

**Authors:** Ying Xin, Jianzeng An, Jia Xu

**Affiliations:** School of History, Anhui Normal University, Wuhu, China

**Keywords:** community, care service, older people, volunteerism, China

## Abstract

**Introduction:**

China has limited formal care services and weak unpaid informal care support for older people, which has caused a care service shortage for them. Voluntary community care services are thus a type of formal care service that aims to meet older people's unmet care needs. However, the continuity of such voluntary community care services is important for the degree to which these unmet care needs of older people can be satisfied. Therefore, this study examines what motivates volunteers to provide voluntary community care services for older people in China. It argues that providing continuous voluntary community care services can be motivated by the interaction of volunteers' internal and external motivations.

**Methods:**

This study employs the grounded theory approach, including open coding, axial coding, selective coding, and saturation testing, and derives the data from 15 semi-structured interviews with volunteers from September to December 2021 in Wuhu, China.

**Results:**

The analysis identifies three internal motivations (altruism, social interaction, and self-fulfillment) and three external motivations (social support, standardized management, and relevant benefits) as well as the interaction between them as factors that impact volunteers' willingness to offer continuous voluntary community care services for older people.

**Discussion:**

The study's findings highlight the impact of continuous volunteering on society, which is significant to provide voluntary community care services for older people. It thus contributes to the development of China's care policy and future care supply services as well as serves as a reference for care development models in other welfare states, particularly in places where both formal and informal care are underdeveloped such as China.

## 1. Introduction

Older people (e.g., those aged over 65 years old) often have an increased need for care services because of their higher frequency of healthcare problems. Most welfare states support formal care services or provide public funding for both homecare and residential care (1). The welfare state's co-funding of such formal care costs usually differs depending on recipients' care needs and the form of care provision in question (2). In addition, the welfare state encourages the development of informal care- which is a type of care that provided by family members to meet older people's unmet care needs- such as the provision of a supporting care allowance for family caregivers (2–4). However, older people in China still have high levels of unmet care needs (4) owing to the lack of informal care resources and insufficient formal care from both institutional and residential care (5). Furthermore, the relatively low level of assistance provided by China's care policy makes care services too expensive for older people to cover by themselves, despite the proportion paid by the state (6, 7).

Against this background, China has developed voluntary community care services to meet older people's unmet care needs (8). Voluntary community care services, a type of formal care service that aims to meet older people's unmet care needs, are organized by the community and financially supported by the municipality. Volunteer organizations are responsible for training volunteers to provide care to older people in the community and supported by municipalities through policy guidance and financial support (9). However, the quality and quantity of voluntary community care services are related not only to the actual realization of a higher quality of life for older people (10) but also to the feasibility of providing this type of care continuously (11). For example, providing voluntary community care services intermittently limits their ability to meet unmet care needs (8). While voluntary community care services can be organized and provided to older people continuously in several communities in China (12), the effectiveness and continuity of providing voluntary community care services are relatively poor in others (8).

Therefore, it is important to analyze what motivates volunteers to provide voluntary community care services and generalize the motivations that impact their willingness to do so. On the one hand, research has shown that internal motivations inspire voluntary activities. For example, the social value created by providing voluntary community care services for older people encourages volunteers to actively participate and enhances the continuity of their service provision (11, 12). Planalp and Trost (13) used the Volunteer Functions Inventory to review the motives of 351 US volunteers and showed that their internal motivations to participate in hospice care volunteering included helping others, improving themselves and their social relationships, and pursuing career goals; of these, helping others was the most important internal motivation. Voluntary services also depend on self-interest motivation (14). On the other hand, the provision of voluntary community care services for older people is directly affected by external factors such as the society and culture (15). For example, charitable contributions and volunteer participation are high in individualistic cultures (16). Previous research presents evidence that social morality and ethical norms form positive motivations behind voluntary behavior (17). Individuals who fully internalize social morality are more willing to help people outside their groups.

It is also interesting to explore what motivates volunteers to provide voluntary community care services continuously. Previous research has explained volunteer participation and retention based on the relationship between motivations and behavioral return [i.e., the satisfaction of motivations; (18)]. If an important motivation is satisfied through volunteering, it is easier for volunteers to continue their activity (19), increase their self-esteem (20), and have a strong incentive to understand older people (21). The more volunteers identify with their role and the more they have high autonomous motivation, the more they are likely to continue volunteering for longer (21, 22). Moreover, the relationship between volunteers and their environment (23), including family support and social interactions during volunteering, impacts the continuity of their voluntary behavior (24, 25). In addition, the social networks that volunteers build during volunteering affect the continuity of the provision of their volunteer services (26, 27). The relationship between volunteers and volunteer organizations thus has an important impact on shaping the continuity of volunteers' behavior (28). The closer volunteers' preferences fit with the incentives provided by volunteer organizations, the higher is their contribution in terms of time and participation (29). Volunteer organizations therefore enable volunteers to both provide volunteer services and realize their value preferences (30).

Rapid demographic aging poses a major challenge to China's health and care systems, as individuals over 65 years account for 13.5% of the country's population (31). However, the Chinese government has not yet developed a comprehensive LTC policy at the national level. The infrastructure of both formal care and residential care facilities remains poorly developed and the service provision differs considerably between rural and urban areas (4). Hence, despite some governmental efforts, a considerable mismatch between the provision of LTC services and demand for such services from older people is presented in practice (32). Therefore, most care-dependent older people still rely on informal care (33). However, family members' abilities to provide care is declining, as it is becoming more difficult for such caregivers (who are usually women) to reconcile their multiple care responsibilities with their work obligations (1, 3). Consequently, the Chinese government is restructuring its LTC system and extending formal care services to address older peoples' rising demand for care and the increasing pressure on families to provide such care; however, it mainly targets older people with limitations to their activities of daily living (34). Furthermore, approximately 50 Chinese cities have run pilot projects to test different LTC policy designs (4, 35).

However, experiences with the current Chinese LTC system and pilot projects have revealed challenges to developing a new LTC policy, particularly the challenge of insufficiently developed formal care provision, declining potential for family members to offer care, and insufficient public support for family caregivers (3, 36). Hence, a gap remains between care services for older people and their unmet care needs. Against this background, the provision of voluntary community care services could bridge this gap to some extent because governments globally are increasingly looking to the voluntary sector as potential welfare providers. According to Zhang and Tian (12), the number of registered volunteers in China reached 217 million by the end of 2021, representing around 1,500 registered volunteers per 10,000 people on average. China's voluntary community care services have also been found to have played an important role in supporting care for older people (12).

It is clear from previous literature that internal and external motivations impact volunteers' capacity to provide voluntary community care services continuously. This study examines what motivates volunteers to provide continuous voluntary community care services for older people in China and whether the interaction between internal and external motivations impacts such voluntary behavior. This study focuses on the provision of voluntary community care services in Wuhu, Anhui province, where volunteering services are roughly equivalent to the national average (12, 37). The Wuhu local government has organized volunteering training courses for volunteers that focus on care services and pandemic governance each year since 2013 (37). Further, it financially supports volunteer organizations that provide voluntary community care services (37).

## 2. Materials and methods

### 2.1. Materials

This study employed semi-structured face-to-face in-depth interviews with 15 volunteers, conducted from September to December 2021. Each of the in-person interviews lasted between 40 and 90 min. The semi-structured interviews used a prepared topic guide as a starting point, which included prompts and open-ended questions. They covered two main topics: (i) the factors that drove volunteers' continued provision of voluntary community care services for older people and (ii) the degree to which internal and external motivations encouraged them to provide care services. Two open-ended questions were included based on the volunteers' planned provision of voluntary community care services for older people in the next 5 years and the extent to which they expected to encourage family members and relatives to provide voluntary community care services. The interviews were concluded when the volunteers began to add no new information.

All the interviews were recorded and later transcribed. Informed consent (both written and verbal) was obtained before the interviews and the confidentiality and anonymity of the participants during the data analysis were ensured. Ethical approval was obtained from Anhui Normal University and this study was conducted in accordance with the ethical principles regarding human experimentation in the Declaration of Helsinki.

All the volunteers were interviewed individually in the meeting room of a community center. After each interview, three authors first sorted the interview records to avoid information loss, refined and summarized the recorded content, and formed concepts that described the motivations of the volunteers to provide voluntary community care services for older people. The transcripts were initially segmented by three authors. Coding was conducted by discussion of each participants response among all three authors; in rare cases when two authors disagreed, the third author acted as arbiter. Through this process, authors selected codes for the analysis of the research question. After assigning codes, all authors participated in the process of placing codes into sub- and superordinate categories. The consistency of the coding by these authors was high and the selected codes and coding results were rechecked by two authors again to avoid bias. Hence, the coding had high reliability. The different motivations that encouraged volunteers to provide voluntary community care services were further discussed by all three of the authors during meetings.

The datasets generated and analyzed during the current study are not publicly available. The anonymized data can be obtained only with the approval of all three authors at the same time as well as the approval of the local government, which provided partial financial support for the current study. Further, Anhui Normal University, which also provided partial financial support for the current study, must provide its approval.

### 2.2. Methods

#### 2.2.1. Participants

The empirical analysis in this study is based on our interviews with 15 volunteers from Wuhu City, Anhui province in China, where the population size and age is considered to be moderate (9). Furthermore, Wuhu City's socioeconomic development is also average compared with other Chinese cities (38). The 15 interviewees were selected from six communities (see Table A1) based on the following inclusion criteria: (i) continuously participate in community volunteering, (ii) have joined at least one volunteer organization, and (iii) have at least 1 year of uninterrupted experience in providing voluntary community care services to older people. Participants were recruited by sending invitations and application forms to both voluntary organizations and communities. We then checked all applications and selected the participants based on our inclusion criteria. Finally, we sent the decision letter to potential participants. Any of the participants could have withdrawn from the study at any time for any reason.

#### 2.2.2. Grounded theory and coding procedure

This study adopted grounded theory (a qualitative approach) to extrapolate the interview data (39). Grounded theory is a relatively scientific and effective method of analyzing data in qualitative research (39–42). The central idea when collecting interview data is the induction and summary of the original data material to reflect the core concepts of a social phenomenon (39). Through repeated data comparisons and coding analysis of the relationship between the concept and data thinking, comparison, analysis, classification, conceptualization, and category, a practical theoretical framework is built (43, 44). Grounded theory does not need a priori conclusions and assumptions based on empirical data to establish a concept (45) and mainly adopts qualitative analysis methods (46, 47). Its main advantage is its ability to compare and analyze interview data in order to propose categories, which has gained this method a good reputation for qualitative data analysis in many research fields (48, 49).

Coding is the most important way to implement grounded theory. The coding procedure has three categories, namely, open coding, axial coding, and selective coding, and saturation testing is used to analyze observational interview data (47). All the codes are derived from the repeated reading of the original data word by word. In this study, all the authors extracted codes in the form of concepts and constantly modified them. Furthermore, a theoretical saturation test is needed after the coding procedure to test the credibility and validation of the interview data (39). Although the number of volunteers used in the saturation test is unlimited in grounded theory (50, 51), the coding results are saturated when interview data are added into the coding model that do not contribute a new concept or conclusion (52, 53). Therefore, only 12 of our 15 volunteers (A01, A02, A03, A04, A05, A07, A08, A09, A10, A12, A13, and A15) were randomly selected for the coding analysis, while the remaining three (A06, A11, and A14) were used as samples for the theoretical saturation test.

## 3. Results

### 3.1. Open coding

Open coding is the first-level coding when conducting grounded theory research (54). In this step, through the repeated reading of the original data word by word, concepts are extracted in the form of codes and constantly modified. This is because the purpose of coding is to further discover the class, and this needs the constant summarization and abstraction of codes to form concepts.

The authors first analyzed the original data and summarized the conditions and influencing factors affecting volunteers' provision of voluntary community care services for older people into the following 17 concepts: sympathy, empathy, social responsibility, relationship construction, group belonging, value realization, beyond self, organizational support, community support, family support, public support, government support, standardized management, industry standards, spiritual benefits, self-development benefits, and material benefits. Table 1 shows the 48 typical representations representing these 17 concepts and explains the connotations of the concepts.

**Table 1:** List of open coding results.

**No**.	**Concept**	**Interview record examples (original material statements)**	**The connotation and interpretation of the concept**
1	C1 Sympathy	A01 At first, I met an old man who had both esophageal cancer and bladder cancer, and I have been volunteering since then.	Having care and compassion for older people is the motivation behind volunteer service.
		A04 Facing the elderly who need surgery but have financial difficulties, watching them when they need to be taken care of and need supplemental financial assistance, I feel disturbed.	
		A13 The most important thing for volunteers is to have time to volunteer for activities with a high level of consciousness and love.	
		A15 For the two elderly people with really poor conditions, volunteers would send them some supplies from time to time. Everyone felt that the elderly needed more help and volunteered.	
2	C2 Empathy	A04 Everyone gets old; so, I hope to form a social atmosphere of caring for the elderly, where the elderly can go now, and we can get care in the future.	From the perspective of older people, they can feel the urgency of their needs, and experience disappointment when they are helpless, which can trigger the motivation of volunteer service.
		A08 Seeing many elderly people in the community, I think of my grandparents; so, I often participate in volunteer activities.	
		A09 People will grow old, and they will need volunteers' help; so, it is necessary for volunteers to act when they still have the ability to help others.	
3	C3 Social responsibility	A08 According to my own ability, I contribute as much as I can and help the elderly in need, and do not consider how much honor I can get.	A prosocial personality and realization of the importance of devoting their time and energy can act as motivation to help older people in the community through voluntary actions.
		A09 In the consciousness of volunteers, volunteer service is selfless dedication and should not be rewarded. We should all have a sense of mutual help.	
		A12 Many of our volunteers like public welfare and like to do volunteer services. They generally want to come to our organization to do good deeds.	
		A15 Society needs everyone to contribute love, and we have the responsibility to build a loving society together.	
4	C4 Relationship construction	A02 Our volunteer organization will regularly carry out service activities for the elderly, in which we can meet old friends in the volunteer team and make friends who have the same interests and values.	By being part of a voluntary organization and voluntary service, they can make friends, build various social connections, and carry out social interaction.
		A07 I got along with this volunteer; so, we often volunteer together.	
		A08 When it was my first time to volunteer, I was brought by a friend. Later, I also organized other friends to volunteer together. In the volunteer activities, our relationship became closer.	
		A10 In participating in volunteer activities, you can meet many friends, including other volunteers, as well as the elderly who receive help.	
5	C5 Group belonging	A02 In each activity, we wear volunteer clothes and serve the elderly as a team, which makes us feel that we have partners.	Take volunteer organizations as the attribution and being a volunteer as a symbol of their own social identity.
		A10 We don't care about the rewards but care about doing something meaningful together.	
6	C6 Value realization	A03 I'm too old to do physical work; but [I do] some simple volunteering to do what I can, [which] makes me feel like I'm needed.	Through the community pension volunteer service, they can devote the time and energy to show that they are useful.
		A05 I can help others and feel valuable.	
		A10 We have a group, most of whom are care workers in the rest home. They have dedication and skills in this field. They want to provide services for elderly people which reflect their own values.	
7	C7 Beyond self	A01 Although I am retired, I can still form a team of volunteers to prove that I can still work.	By forming volunteer organizations and participating in voluntary actions, they get the opportunity to find “another valuable self” and surpass the existing social identity.
		A03 I had a job before retirement. After retirement, I want to find myself something to do. Volunteering to provide care services is the thing that I prefer.	
		A05 I take a break from my work and life circle in my spare time, which is also one reason why I participate in the volunteer team.	
8	C8 Organizational support	A01 The volunteer organization handed over the burden of celebrating the collective birthday for the elderly to me, which is the trust of the volunteer organization, and I must do a good job to be worthy of the organization's trust in me.	Volunteer organizations set up platforms and raise resources to provide support for volunteers to participate in community care service for older people.
		A04 The volunteer organizations I participate in have strong action ability. We volunteers help each other and provide services for the elderly together, and the action is much more convenient and effective.	
		A08 Volunteer organizations carry out household skills, work skills, and project development training, and supervise the progress of the service project.	
		A12 Every time we carry out volunteer service activities, volunteer organization managers lead us.	
9	C9 Community support	A02 The community neighborhood committee is very supportive of conducting voluntary service activities for the elderly in the community, providing support including service activity venues, information about the elderly, and some necessary materials.	The community neighborhood committee provides property, information, prestige, and other support to volunteer organizations and volunteers.
		A10 Community neighborhood committees often recommend volunteers to volunteer organizations to promote the expansion of the volunteer team.	
		A15 The community neighborhood committee recommended the service deeds of volunteer organizations to the media for publicity, and also publicized them within the community, which enhanced the social recognition of volunteer organizations and volunteers.	
10	C10 Family support	A13 My children are very supportive of me taking part in volunteer service activities.	Family members understand and support voluntary participation behavior.
		A15 At first, I volunteered, and then my husband joined our volunteer organization.	
11	C11 Public support	A03 Our volunteer activities are often reported by the media, and the volunteer team is growing.	The public supports and praises the volunteers and their participation behavior.
		A09 After hearing about our volunteer action deeds, some enterprises or individuals will donate some items to support us to provide services for older people.	
12	C12 Government support	A05 Many of the government's elder-care policies mention volunteers, indicating that volunteers are important for pension services.	The government encourages and supports voluntary activities through policy guidance and resource support.
		A10 Many of our volunteering activities are government projects, and we receive financial support.	
		A15 In the selection of various moral models, the government also included our volunteers in the selection scope, and gave us a good reputation and other awards, which shows that the government attaches great importance to volunteers.	
13	C13 Standardized management	A04 Not everyone can volunteer. Those who are ready to join us must be assessed, and those who do not meet the requirements of volunteer organizations should be eliminated.	The standardized management and formal operation of volunteer organizations promote volunteers to provide community care services for older people.
		A08 Voluntary organizations have various management systems within them, involving codes of conduct, transportation subsidies, service processes, etc.	
		A10 Within voluntary organizations, there are assessments of volunteer service performance.	
14	C14 Industry standard	A09 Although we are voluntary and grassroots, our state has many norms of voluntary behavior, and the grassroots governments and community neighborhood committees will also manage the voluntary service work within the community.	The government and community neighborhood committees manage and standardize the voluntary behavior and promote the occurrence of voluntary behavior.
		A15 Grassroots governments and community neighborhood committees do not allow voluntary organizations to develop arbitrarily but have many management systems; they will also evaluate and manage the values, abilities, and service effectiveness of volunteer organizations and volunteers.	
15	C15 Spiritual benefits	A07 For my volunteer work, I have won a variety of government moral honors, which are also great encouragements to me.	Emotional and psychological motivation for volunteers to participate in community care service for older people.
		A09 At the end of the year, volunteer organizations will select excellent models according to the service duration, service performance, and service contribution value, and present awards at the community party, which is undoubtedly an honor.	
16	C16 Self-development benefits	A05 The government arranges a series of volunteer trainings to provide service skills, which is not only conducive to improving service efficiency, but also improves the ability of our volunteers.	Self-development benefits for volunteers to participate in community care service for older people.
		A08 Government departments often organize some professional theme training on social work, including offline training and online courses, to improve the service ability of volunteers in multiple ways.	
17	C17 Material benefits	A12 Volunteer organizations will give a certain subsidy to the volunteers.	Material motivation for volunteers to participate in community care service for older people.

### 3.2. Axial coding

The aim of the axial coding stage in this study was to overcome the drawbacks of the open coding stage owing to the unclear relationships found among the 17 concepts. Hence, to further understand the correlations among the 17 concepts, we needed to analyze the logic behind the provision of voluntary community care services for older people. Further axial coding (also known as associated coding) of the open coding results was also required. Therefore, the 17 concepts extracted from the open coding were classified into two main categories and six subcategories, as shown in Table 2.

**Table 2:** Axial coding results.

**Main category** ** (Two items)**	**Subcategory** ** (Six items)**	**Concept(s)** ** (17 items)**
F1 Internal motivations	f1 Altruism	C1 Sympathy; C2 Empathy; C3 Social responsibility
	f2 Social interaction	C4 Relationship construction; C5 Group belonging
	f3 Self-fulfillment	C6 Value realization; C7 Beyond self
F2 External motivations	f4 Social support	C8 Organizational support; C9 Community support; C10 Family support; C11 Public support; C12 Government support
	f5 Standardized management	C13 Standardized management; C14 Industry standards
	f6 Relevant benefits	C15 Spiritual benefits; C16 Self-development benefits; C17 Material benefits

### 3.3. Selective coding

The aim of the selective coding stage in this study was to systematically mine the logical correlations and summarize the typical relationships among these categories (55), as shown in Table 3. We surmised that the continuous provision of voluntary community care services for older people occurs owing to both internal and external motivations. For example, volunteer A01 (September 8, 2021) volunteered because she is activated by both “altruism” and “self-fulfillment” (internal motivations) as well as “social support” (external motivation). Moreover, we found that these internal and external motivations do not need to be possessed at the same time. However, at least one motivation from both groups must be present for continuous voluntary participation to occur. This suggests that the interaction between internal and external motivations has a great impact on stimulating volunteers' continuous provision of voluntary community care services. We thus used saturation testing to verify the credibility of this finding.

**Table 3:** Selective coding results.

	**Volunteer number**	**Internal motivations**			**External motivations**		

**No**.		**Altruism**	**Social interaction**	**Self-fulfillment**	**Social support**	**Standardized management**	**Relevant benefits**
1	A01	**√**		**√**	**√**		
2	A02		**√**		**√**		
3	A03			**√**	**√**		
4	A04	**√**			**√**	**√**	
5	A05			**√**	**√**		**√**
6	A07		**√**				**√**
7	A08	**√**	**√**		**√**	**√**	**√**
8	A09	**√**			**√**	**√**	**√**
9	A10		**√**	**√**	**√**	**√**	
10	A12	**√**			**√**		**√**
11	A13	**√**			**√**		
12	A15	√			**√**	**√**	**√**

√ Indicates the motivations included in the volunteers' statement. A06, A11, and A14 were selected for saturation testing.

### 3.4. Saturation testing

As noted above, we randomly selected three volunteers (A06, A11, and A14) for the saturation testing (volunteer A06: September 8, 2021; volunteer A11: September 7, 2021; volunteer A14: November 15, 2021). The test results conformed to the typical relationship structure without finding new concepts/categories or new connections among the categories, which confirmed that the open coding, axial coding, and selective coding had reached saturation and that the interaction between the internal and external motivations was credible (see Table A2). The decision-making of rational individuals is often related to their values and the external conditions (56). Hence, this study argues that the continuous provision of voluntary community care services for older people depends not only on volunteers' inner value judgment of volunteering activities but also on the institutional rules, social support, and other external conditions.

## 4. Discussion

According to our coding analysis, we found six motivations (three each for internal and external motivations) that affect the provision of voluntary community care services for older people and its duration. These six motivations also explain the interaction between internal and external motivations and its impact on the continuity of providing voluntary community care services for older people.

### 4.1. Internal motivations and the continuity of providing voluntary community care services for older people

This study argues that voluntary action is driven by three internal motivations: altruism, social interaction, and self-fulfillment. First, altruism stands opposite to egoism, as it involves voluntarily helping others (57). Altruistic behavior does not have spiritual or material benefits as its primary purpose. It occurs when people know that providing their materials, time, and energy will not guarantee them any return. Actions taken based on altruism uphold moral logic—whether to act is decided according to ethical principles such as compassion, humanity, love, and justice, while material benefits and costs are ignored (58). Hence, voluntary behavior does not pursue self-interest, does not act on market principles, and has a natural correlation with altruistic motivation (volunteer A10: September 8, 2021). For example, volunteers help others voluntarily and expect no return, and society needs such people to provide voluntary community care services for older people in China (volunteer A08: September 8, 2021). The fact that such volunteers are willing to contribute their time and energy, rather than pursue direct returns, reflects their sense of social responsibility and community cohesiveness. Even without external rewards and motivations, people will take the initiative to help when they see a need for their services (volunteer A08: September 8, 2021). In conclusion, altruism prompts volunteers to help older people in need.

The second important motivation for volunteers' continuous provision of voluntary community care services for older people is social interaction. Individuals may feel lonely and scared without a social network. Volunteers find their lives to be more meaningful and interesting when volunteering, for example by making friends that have the same values (volunteer A02: September 8, 2021). Therefore, integrating into the community, entering society, and building social networks have become important for individuals deciding to provide voluntary community care services continuously. People want to build relationships and play a role in a team in order to avoid loneliness as well as gain social recognition, membership, and group affiliation (volunteer A11: September 7, 2021). Volunteers can communicate with the older people they assist, exchange information, develop communication skills, share experiences with other volunteers, build friendships with people who share their interests and values, expand their social networks, and reduce the sense of social alienation (volunteer A02: September 8, 2021).

Moreover, volunteer organizations, as a community network, provide volunteers with a sense of group ownership; volunteers who support each other and cooperate in the service process then also cultivate a sense of group ownership (volunteer A12: September 7, 2021). By serving the same volunteer organization, volunteers can deeply understand the close connection between their personal development and society, which enhances their sense of social belonging. Thus, the voluntary community care services provided by volunteers from volunteer organizations can help build their social belonging in the form of both tangible social belonging such as the circle of volunteers and volunteer organizations (volunteer A02: September 8, 2021) and the invisible social belonging existing in the form of ideas such as shared volunteerism and common altruistic values (volunteer A02: September 8, 2021). These two types of social belonging increase the frequency and depth of interpersonal communication at different levels, which can also enhance the trust, assistance, and reciprocity among volunteers (volunteer A08: September 8, 2021). It also increases the tendency for volunteers to take common actions such as providing continuous voluntary community care services for older people.

Finally, many volunteers are also motivated by their need for self-fulfillment, which is the third internal motivation. Self-fulfillment, the highest psychological need, occurs only when the needs pertaining to physiology, safety, belonging and love, and self-esteem are fulfilled (59). Self-implementers tend to pursue the values of truth, charity, and vitality rather than economic factors (60). The need for self-fulfillment motivates volunteers, in particular retiree volunteers, to start and continue to provide voluntary community care services. Further, their continuous willingness to volunteer strengthens as their satisfaction of self-fulfillment increases (volunteer A05: September 7, 2021). For example, retirees join volunteer organizations and provide voluntary community care services to older people with care needs because they want to show that they are still dynamic and useful to society (volunteer A03: September 7, 2021). Providing help to older people with care needs in their spare time for free indicates their social efficiency, demonstrates their social value, and confirms their feelings of being needed (volunteer A01: September 8, 2021).

### 4.2. External motivations and the continuity of providing voluntary community care services for older people

The continuity of volunteering behavior is also affected by three external motivations, namely, social support, standardized management, and relevant benefits. First, this study argues that social support, including voluntary organizations' support, community support, family support, government support, and public support, is an important external motivation for promoting continuity in the provision of voluntary community care services for older people. In detail, volunteer organizations support this provision by promoting volunteering and filial piety, activating the altruistic motivation of volunteers, training multiple volunteers to achieve the service goals set by those organizations, laying the resource foundation for the service, designing service plans, and implementing effective management techniques (e.g., volunteer A13: September 7, 2021; volunteer A08: September 8, 2021). When these organizations' work raises volunteers' satisfaction and enjoyment, volunteers are happy to continue to provide voluntary community care services (volunteer A08: September 8, 2021; volunteer A04: September 8, 2021). Support from the community also promotes their provision of voluntary community care services, with many volunteers even recommended by community committees. The community helps design volunteers' service projects and offers partial financial support for their involvement (volunteer A15: September 8, 2021). In addition, family support for volunteers is important to promote the provision of voluntary community care services for older people, especially when family members are also willing to provide such support, as this is when the continuity of voluntary actions greatly increases. Moreover, government support is important for volunteers to provide voluntary community care services (volunteer A14: November 15, 2021). The government is one of the main financial resources for voluntary community care services projects for older people. For example, the Chinese government has recently provided a reward for volunteers named as “good citizens” for the important work they perform. Two of our volunteers mentioned that the spirit of voluntary action is derived from government recognition and support (volunteer A12: September 7, 2021; volunteer A05: September 7, 2021). In addition, public support is significant since it offers public financial donations toward the provision of voluntary community care services for older people. More than half of our volunteers asserted that volunteer organizations and their services would be unsustainable without charitable donations (volunteer A09: September 8, 2021).

Second, standardized management is another necessary external motivation that motivates volunteers to provide voluntary community care services. Volunteers join volunteer organizations and actively provide voluntary community care services for older people because they trust these organizations (volunteer A04: September 8, 2021) owing to their standardized management and regulated principles. These volunteer organizations are, in principle, formal organizations with structured systems and clear rules in place, which attract volunteers' trust and sense of belonging. The volunteer organizations to which most of our interviewees belonged have strict personnel management systems, clear project management systems, relatively strong financial management systems, clear service quality assessment criteria, performance evaluation standards, and motivational systems to encourage volunteers with clear goals (volunteer A07: November 15, 2021). Besides, many volunteers in the same volunteer organization are friends, neighbors, colleagues, and even family members, which makes it easy for them to develop trust and feel happy when they cooperate (volunteer A02: September 8, 2021; volunteer A07: November 15, 2021). Municipality and community committees' issuance of administrative regulations on volunteer organizations' services and assessment criteria as well as the social rights of volunteers also enhance the standardization of the management practices of volunteer organizations (volunteer A07: November 15, 2021). Volunteers experience a decreased sense of service anxiety and concern about unforeseen circumstances under clear management regulation, while their enthusiasm for providing care services for older people increases (volunteer A08: September 8, 2021; volunteer A12: September 7, 2021). Therefore, standardized management is an external motivation that contributes to the continuous provision of voluntary community care services for older people.

Third, volunteers obtain relevant benefits when providing voluntary community care services for older people, including spiritual benefits, self-development benefits, and material benefits. First, for spiritual benefits, volunteers might feel valuable by offering care services, which increases their self-evaluation and confidence (volunteer A05: September 7, 2021). Second, for self-development benefits, volunteers might actively strive to realize their self-worth, such as by enjoying friendships and social recognition, as well as gain social responsibility and develop a positive outlook on life (volunteer A09: September 8, 2021). Moreover, volunteer organizations usually arrange various training programs for volunteers to enhance their communication skills, management skills, and social work skills, thereby increasing their service efficiency (volunteer A05: September 7, 2021). Third, for material benefits, volunteers might be financially rewarded by volunteer organizations, the community, and the local government for being “good citizens” (volunteer A06: September 8, 2021). For example, many volunteer organizations have established volunteer service points systems, which provide volunteers with service points based on the quality and frequency of their provision of care services in the city. These points can be used in charity supermarkets to purchase items such as food and drink. Older volunteers can also buy for-profit long-term voluntary community care services with these points (volunteer A12: September 7, 2021). These benefits can raise the possibility of providing continuous voluntary community care services for older people.

### 4.3. Interaction of internal and external motivations and its impact on the continuity of providing voluntary community care services for older people

The correlation between these six internal and external motivations is essential. On the one hand, we found that in the cases when all three internal and three external motivations are present in volunteers, it aids the continuity of their provision of voluntary community care services for older people. We argue that if volunteers are only driven by either internal or external motivations, their voluntary behavior may occur occasionally instead of continuously and their care service provision may be fragmented instead of systematic. On the other, we found that voluntary community care services can be provided continuously when volunteers have only one or two motivations from the internal and external motivation categories. This indicates that there is no direct correlation between the three motivations of each motivation category and that the roles of internal and external motivations are not interchangeable. Instead, this shows that internal and external motivations affect continuous voluntary behavior differently and that the role of these two motivation categories cannot replace one another. Thus, although the analysis of the interview data shows that the continuous provision of voluntary community care services for older people requires both internal and external motivations, it is not necessary that all six motivations are present.

According to our interview data, the desire to volunteer to provide voluntary community care services for older people is determined partly by internal motivations, as they frame volunteers' social and organizational roles. In short, a person may be altruistic under certain external motivations; however, this does not correspond to the continuity of providing voluntary community care services. Hence, although internal motivations can promote volunteers' willingness to provide voluntary community care services for older people, the provision of such care services in practice also needs the support of institutions such as volunteer organizations. It seems that external motivations provide volunteers with a basic guarantee of being able to supply voluntary community care services. Therefore, internal and external motivations are necessary but not sufficient separately to ensure the continuous volunteering of voluntary community care services. Only through the simultaneous presence of external and internal motivations and the interaction between them can the continuous provision of voluntary community care services for older people be promoted. Consequently, internal and external motivations interact to help provide continuous voluntary community care services for older people. The behavior of volunteers that provide voluntary community care services for older people differs mainly because volunteers experience different motivation interactions.

This study contributes to the fields of community psychology as well as personality and social psychology since it deals with the development of volunteering through different types of motivations. It helps clarify the continuous provision of care services for older people to improve their quality of life while meeting their unmet care needs. However, while this study is a good reference for other countries organizing voluntary community care services, it also has limitations concerning the number of interviews and restricted coverage of older people, which limit the generalizability of its results. This study is also limited since it only focuses on one region in China and lacks broader research in different regions at diverse stages of socioeconomic development.

Future research should focus on discovering whether the lack of interaction between internal and external motivations is driven by the lack of value support and meaning construction or the lack of structural support. In addition, more factors could be included in the analysis of internal and external motivations and their impact on the continuity of providing voluntary community care services for a broader group of people such as vulnerable populations. Future research could also compare continuous voluntary behavior across China.

## Data availability statement

The raw data supporting the conclusions of this article will be made available by the authors, without undue reservation.

## Ethics statement

The study protocol was approved by the Ethic Committee of Anhui Normal University of China (Approval No. AHNU-ET2022046). The patients/participants provided their written informed consent to participate in this study. Written informed consent was obtained from the individual(s) for the publication of any potentially identifiable images or data included in this article.

## Author contributions

YX collected the interview data on the community care services and analyzed and interpreted the interviews with JA. JA and JX contributed to the writing, structure, and design of the manuscript. All authors read and approved the final manuscript.

## Appendix

**Table A1:** Information on the volunteers.

**No**.	**Volunteer number**	**Sex**	**Age (years)**	**Position**	**Volunteer experience in community care service for older people (years)**	**Evaluated by volunteer organization (times)**	**Community**
1	A01	Female	78	Head of the volunteer organization	10	50	Hongqiao Community (H.Q.)
2	A02	Female	30	Volunteer	3	6	Hongqiao Community (H.Q.)
3	A03	Female	65	Head of the volunteer organization	7	35	Qingyuan Community (Q.Y.)
4	A04	Female	20	Volunteer	1	2	Hongqiao Community (H.Q.)
5	A05	Female	27	Volunteer	2	4	Jiuzi Community (J.Z.)
6	A06	Female	70	Volunteer	3	6	Rulin Xiyuan Community (R.X.)
7	A07	Male	35	Volunteer	3	6	Xuri Tiandu Community (X.T.)
8	A08	Male	40	Head of the volunteer organization	14	61	Central City Community (C.C.)
9	A09	Male	63	Volunteer	8	16	Jiuzi Community (J.Z.)
10	A10	Male	30	Volunteer	2	4	Central City Community (C.C.)
11	A11	Female	28	Volunteer	4	8	Central City Community (C.C.)
12	A12	Male	50	Volunteer	5	10	Qingyuan Community (Q.Y.)
13	A13	Male	75	Head of the volunteer organization	10	50	Qingyuan Community (Q.Y.)
14	A14	Female	30	Volunteer	4	8	Xuri Tiandu Community (X.T.)
15	A15	Female	69	Volunteer	5	10	Rulin Xiyuan Community (R.X.)

Volunteer numbers are randomly determined.

**Table A2:** Saturation testing.

**No**.	**Volunteer number**	**Internal motivations**			**External motivations**		

		**Altruism**	**Social interaction**	**Self-fulfillment**	**Social support**	**Standardized management**	**Relevant benefits**
1	A06	√	√		√		
2	A11	√				√	
3	A14		√				√

A06, A11, and A14 all supported the conclusion that the continuous provision of voluntary community care services for older people is influenced by two factors and both have internal and external motivations, which is consistent with the case of the other volunteers. The test results conformed to the typical relationship structure without finding new concepts and categories or any new connections between the categories.
